# Continuous Monitoring of Hydric Deformation in Macigno Sandstone After Thermal Conditioning

**DOI:** 10.3390/ma19183866

**Published:** 2026-09-11

**Authors:** Marco Lezzerini, Stefano Pagnotta, Maria Pia Riccardi

**Affiliations:** 1 Department of Earth Sciences, University of Pisa, Via S. Maria 53, 56126 Pisa, Italy; 2 Consorzio Interuniversitario Nazionale per la Scienza e Tecnologia dei Materiali, Consorzio Interuniversitario Nazionale per la Scienza e Tecnologia dei Materiali (INSTM), 50121 Firenze, Italy; 3 Scuola di Restauro, Academy of Fine Arts in L’Aquila, Via L. da Vinci 6b, 67100 L’Aquila, Italy; s.pagnotta@abaq.it; 4 Department of Earth and Environmental Sciences, University of Pavia, Via Ferrata 1, 27100 Pavia, Italy; mariapia.riccardi@unipv.it

**Keywords:** Macigno sandstone, hydric deformation, continuous monitoring, thermal conditioning, water absorption, ultrasonic pulse velocity, bedding anisotropy

## Abstract

**Highlights:**

- Specimens were monitored throughout the sequential thermal-conditioning pathway.
- Hydric deformation remained higher normal to bedding than in the bedding-parallel direction.
- At 500 °C, hydric deformation decreased by 67% (MTO) and 76% (MTP).
- Water absorption increased as hydric deformation decreased.
- Ultrasonic pulse velocity decreased moderately, consistent with fabric modification.

**Abstract:**

Hydric deformation can contribute to progressive damage in building stones, particularly when moisture-induced strains interact with pre-existing fabric anisotropy and thermally induced microstructural changes. Six Macigno sandstone prisms (20 mm × 20 mm × 200 mm), three with the longitudinal axis orthogonal to bedding (MTO) and three parallel to bedding (MTP), were examined after drying at 60 °C and after sequential thermal conditioning at 350 and 500 °C. At each stage, hydric deformation was continuously monitored during 8400 min of water immersion, and at the end of each test, water absorption and ultrasonic pulse velocity were measured on the samples. Mean final hydric deformation in the 60 °C reference state was 0.54 mm m^−1^ for MTO and 0.44 mm m^−1^ for MTP. Along the sequential conditioning path, it decreased to 0.18 and 0.10 mm m^−1^, respectively, at 500 °C, whereas water absorption increased from about 0.42 to 0.58–0.59 wt.%. Ultrasonic pulse velocity decreased from 4347 to 3915 m s^−1^ for MTO and from 4770 to 4437 m s^−1^ for MTP. Bedding-related anisotropy persisted, while water absorption and hydric deformation followed divergent trends. Continuous acquisition further showed that the general temporal pattern of rapid initial deformation followed by a more gradual approach to a near-stable response was preserved after thermal conditioning, although the initial deformation rate decreased systematically.

## 1. Introduction

Environmental fluctuations in moisture and temperature can induce dimensional changes in sedimentary rocks used as building stones. Although these strains are generally small, repeated expansion and contraction may generate local stresses, promote microstructural deterioration, and contribute to long-term durability loss [1,2,3,4]. Quantitative characterization of moisture-induced deformation is therefore relevant for the assessment of natural stones exposed to changing environmental conditions.

In the present study, hydric deformation denotes the unconstrained dimensional strain induced by wetting of an initially dry rock. It reflects the collective response of the pore structure, mineral–water interactions and rock fabric during water uptake and should be distinguished from deformation generated by externally imposed pore pressure or hydraulic gradients. In sandstones, hydric deformation depends on the combined influence of mineralogical composition, pore structure, cement distribution, and rock fabric [2,3,4,5]. Particular attention has been given to clay-bearing sandstones because hydration and dehydration of clay-rich domains can generate measurable dimensional changes and, under constrained conditions, damaging stresses [5,6,7,8,9,10]. Experimental studies have shown that even relatively small amounts of swelling-active clay minerals can influence the macroscopic response of sandstone, depending on their mineralogy, distribution within the rock framework, and interaction with pore water [5,7,10]. Wangler and Scherer [5], in particular, identified intracrystalline swelling as an important mechanism in clay-bearing sandstones, whereas Jiménez-González et al. [10] demonstrated the important role of clay-bearing domains in the physico-mechanical response of sandstone during wetting and drying.

Fabric anisotropy provides an additional control on hydric deformation. Preferential orientation of phyllosilicates and other fine-grained components may produce different strain magnitudes parallel and orthogonal to bedding, even when the total quantity of absorbed water is similar [7,9]. This distinction is important because water uptake and dimensional deformation represent related but not necessarily equivalent aspects of the interaction between stone and moisture.

Thermal exposure can further modify sandstone behavior by altering pore structure, grain-boundary contacts, and microstructural continuity. Experimental thermo-hydric studies have shown that temperature and moisture act jointly in the weathering response of building stones [11], while investigations of heated sandstones have documented temperature-dependent changes in microstructure and crack development [12,13]. Micro-computed tomography observations, for example, provide direct evidence that thermal treatment can modify the internal discontinuity network of sandstone [13]. Such modifications may increase the amount of water-accessible pore space while simultaneously changing the way in which absorbed water interacts with deformation-active mineral domains. It is important to distinguish the thermal modification of the stone during heating from the subsequent moisture-induced response measured after cooling. In the present experimental protocol, thermal conditioning represents the prior modification of the material, whereas hydric deformation is measured subsequently during water immersion. The observed hydric response should therefore be interpreted as the response of a thermally modified sandstone interacting with water, rather than as a direct deformation effect occurring during heating.

The possible relationship between increased water accessibility and subsequent hydric deformation therefore deserves particular attention. Greater water absorption after heating cannot necessarily be assumed to produce greater moisture-induced strain. Hydric deformation depends not only on the total quantity of absorbed water but also on where that water is accommodated and whether it interacts with mineral domains capable of generating dimensional change [5,10].

From an engineering perspective, thermally affected rocks may also occur in subsurface and energy-related environments where temperature variations coexist with fluid circulation. In such contexts, changes in pore accessibility, fluid uptake and mechanical continuity may influence the subsequent response of the rock. The present study focuses specifically on the moisture-induced dimensional response of building sandstone, rather than on geothermal reservoir behavior [14].

The Macigno Formation of the Northern Apennines consists predominantly of quartz–feldspathic turbiditic sandstones interbedded with pelitic layers [15,16]. In the Matraia area of north-eastern Tuscany, Macigno sandstone has historically been exploited as a dimension stone [15]. The Matraia sandstone is a compact quartz–feldspathic arenite characterized by low porosity, calcite-bearing cementation, and a measurable phyllosilicate component [17,18,19,20]. Previous mineralogical investigations have identified an assemblage dominated by illite and chlorite, with subordinate chlorite-smectite mixed-layer phases and locally corrensite [8,9]. These phases, together with the preferential orientation of phyllosilicate-rich domains, make Macigno sandstone a suitable material for investigating bedding-dependent hydric deformation.

Previous works on Macigno sandstone have demonstrated relationships among mineralogical composition, water absorption, and hydric dilatation [8,9]. However, less information is available on how the hydric response of the same specimens evolves through successive thermal-conditioning stages. A repeated-measurement approach is particularly useful in this context because it limits the influence of natural specimen-to-specimen variability and permits treatment-induced changes to be followed directly.

In the present study, six prismatic specimens of Macigno sandstone were therefore followed after drying at 60 °C and after sequential thermal conditioning at 350 and 500 °C. Three specimens were oriented orthogonal to bedding (MTO) and three parallel to bedding (MTP). Hydric deformation was continuously monitored during water immersion using the dilatometric apparatus previously developed for linear hydric-deformation measurements [21], while water absorption and ultrasonic pulse velocity were determined on the same specimens at each conditioning stage.

The objectives were to (i) characterize the continuous hydric-deformation response of Macigno sandstone and its bedding-related directional dependence; (ii) determine how sequential thermal conditioning modifies hydric deformation in the same specimens; and (iii) compare the evolution of hydric deformation with changes in water absorption and ultrasonic pulse velocity. Particular attention was given to whether increased water uptake after sequential thermal conditioning is accompanied by increased dimensional deformation or whether the two responses show progressively divergent evolution.

Continuous monitoring provides information beyond conventional endpoint measurements because it resolves not only the final magnitude of hydric deformation but also its temporal evolution and approach to a near-stable condition. By combining this information with water absorption and ultrasonic pulse velocity measured on the same specimens, the experimental design allows changes in water uptake, deformation response, and elastic properties to be compared directly along the sequential thermal-conditioning pathway.

## 2. Materials and Methods

### 2.1. Materials and Specimen Preparation

Macigno sandstone was collected from the active Matraia quarry (NE Tuscany, Italy). Fresh, unweathered material was selected by excluding weathered zones, veins, and macroscopic discontinuities.

According to previous quantitative mineralogical characterization of Macigno sandstone from the Matraia quarry [17], the investigated material is a compact quartz–feldspathic arenite and contains approximately 8.5 vol.% phyllosilicates and 5.6 vol.% calcite. Previous mineralogical studies on Macigno sandstones from north-western Tuscany documented illite and chlorite, with subordinate chlorite–smectite mixed-layer phases and locally corrensite [8,9].

Six prismatic specimens measuring approximately 20 mm × 20 mm × 200 mm were prepared using diamond saws under continuous water cooling. Three specimens were cut orthogonal to bedding (MTO; MTO-1 to MTO-3) and three parallel to bedding (MTP; MTP-1 to MTP-3).

The same six specimens were followed throughout the complete experimental sequence. They were first characterized after drying at 60 °C to constant mass and were subsequently thermally conditioned at 350 °C and 500 °C. Thus, measurements obtained at the three conditioning stages represent repeated observations on the same specimens rather than independent specimen groups.

Before the reference measurements, specimens were oven-dried at 60 °C until constant mass, defined as a mass variation of less than 0.1% over 24 h.

### 2.2. Sequential Thermal Conditioning

Following characterization in the reference condition after drying at 60 °C to constant mass, the same six prismatic specimens were subjected sequentially to thermal conditioning at 350 and 500 °C. The conditioning temperatures of 350 and 500 °C were selected to represent two progressively more severe levels of thermal exposure within the temperature range in which physical and microstructural changes have been reported for sandstones, while keeping the maximum treatment temperature below the α–β quartz transition at approximately 573 °C, at atmospheric pressure [12,13]. After completion of the water-absorption and hydric-deformation measurements at each conditioning stage, the specimens were re-dried at 60 °C in a natural-convection oven to constant mass before the subsequent thermal-conditioning step. Because the same specimens were subjected sequentially to water immersion, re-drying, and thermal conditioning, the measurements at 60, 350, and 500 °C represent within-specimen trajectories along a cumulative experimental pathway rather than independent treatment groups. In the absence of a separate control set subjected to repeated wetting–drying cycles without heating, a contribution from measurement history cannot be completely separated from the effects associated with increasing conditioning temperature.

At each conditioning step, specimens were heated in a muffle furnace (Carbolite Furnaces CSF 1200, Carbolite Gero Ltd., Hope Valley, UK) to the target temperature and maintained at that temperature for 2 h. They were then allowed to cool in a desiccator containing silica gel until room temperature was reached. After cooling, the specimens were weighed, and the resulting mass was used as the dry reference mass for the subsequent determination of water absorption. Importantly, hydric deformation was not measured during heating. After each thermal-conditioning step, the specimens were cooled to room temperature before being subjected to water-immersion measurements. Thus, the measured hydric deformation represents the moisture-induced response of the sandstone after thermal conditioning. Ultrasonic pulse velocity was measured before and after the subsequent water-absorption and hydric-deformation tests.

The experimental design therefore consisted of a repeated-measurement sequence in which the same three MTO and three MTP specimens were characterized after drying at 60 °C and after sequential thermal conditioning at 350 and 500 °C. At each stage, ultrasonic pulse velocity, water absorption, and continuous hydric-deformation measurements were performed on the same specimens.

**Figure 1:**
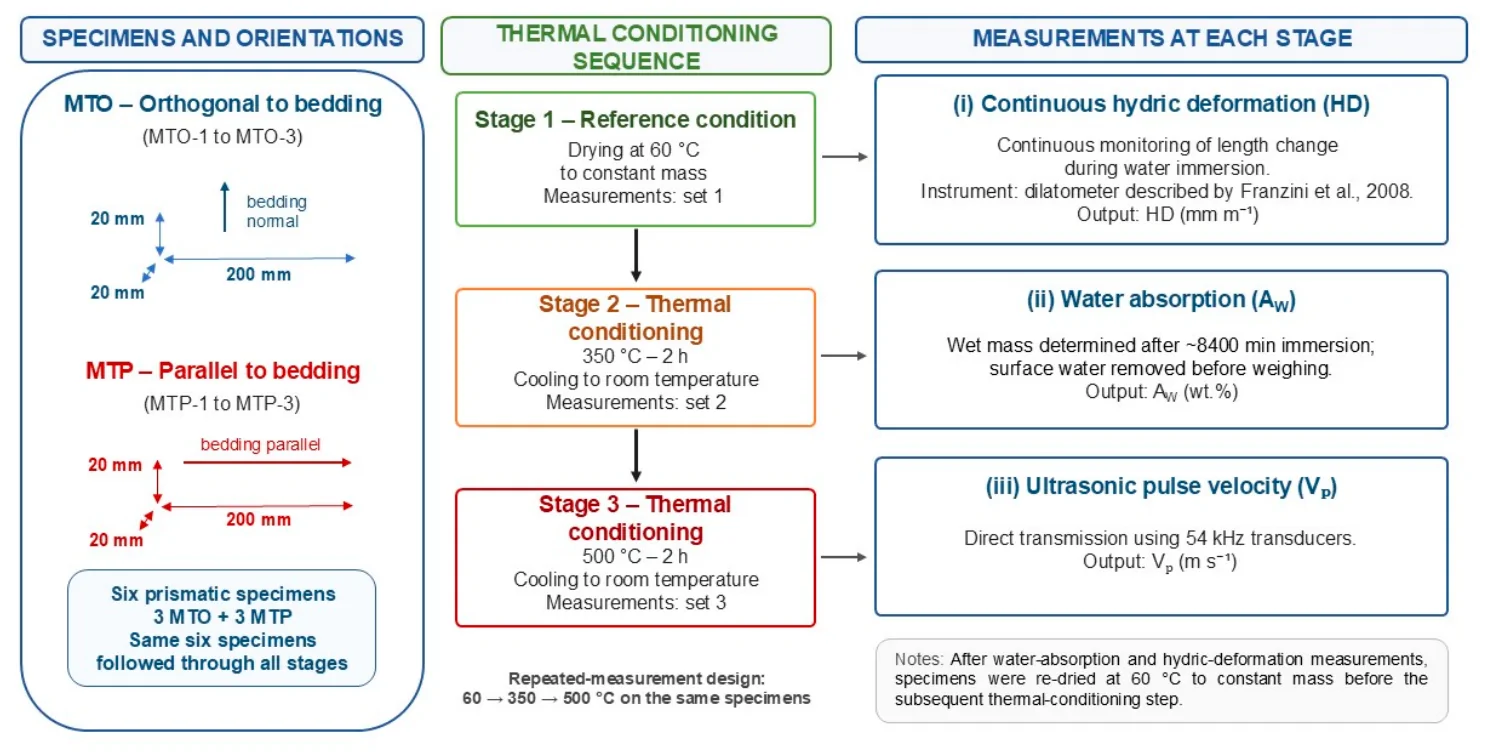
Experimental design and workflow. Experimental sequence adopted for the six Macigno sandstone prismatic specimens. Three specimens were oriented orthogonal to bedding (MTO) and three parallel to bedding (MTP). The same specimens were characterized after drying at 60 °C and after sequential thermal conditioning at 350 and 500 °C. At each stage, ultrasonic pulse velocity, water absorption, and continuous hydric deformation during water immersion were determined using the dilatometer described by Franzini et al. [21].

The experimental sequence and repeated-measurement design are summarized in Figure 1.

### 2.3. Continuous Hydric-Deformation Measurements

Hydric deformation was measured using the dilatometric apparatus previously developed for linear hydric-deformation measurements in rocks [21].

The specimen was positioned between adjustable supports, and longitudinal length changes were transferred to a digital comparator (Mitutoyo 543-250B with RS-232 output, Mitutoyo Corporation, Kanagawa, Japan) through a rocking-arm system providing threefold mechanical amplification. The comparator resolution was 0.001 mm, corresponding to a theoretical displacement sensitivity of approximately 0.0003 mm.

After stabilization and zeroing of the comparator, dry specimens were fully immersed in distilled water maintained at 20.0 ± 0.1 °C using a cryostat (Lauda Proline RP 1840, LAUDA DR. R. WOBSER GMBH & CO. KG, Lauda-Königshofen, Germany). The specimens were immersed under atmospheric pressure, without externally imposed hydraulic pressure or controlled hydraulic gradients. The measured dimensional changes therefore represent the unconstrained hydric response of the specimens during water uptake. Length changes were recorded continuously for approximately 8400 min (about 6 days), with a sampling interval of 10 min, using custom-developed software (V1.0) for serial-port data acquisition. The common observation period of approximately 8400 min was selected to extend well beyond the rapid initial deformation stage and to include the near-stable portion of the response for all specimens. Final hydric deformation was defined as the mean value calculated over the final 30 min of immersion.

To characterize the temporal evolution of hydric deformation, additional kinetic parameters were determined for each individual deformation curve. The initial deformation rate was calculated by linear regression over the first 120 min of immersion. This interval was selected to represent the early deformation stage while providing a sufficient number of acquisition points for a robust estimate of the initial trend. The characteristic times t50 and t90 were defined as the times required to reach 50% and 90%, respectively, of the final near-stable deformation (HDfinal). These characteristic times were obtained by linear interpolation between consecutive measurement points.

Hydric deformation (*HD*) was calculated from the change in specimen length relative to the initial dry length and expressed in mm m^−1^:

$$HD\left(t\right)=\frac{L\left(t\right)-L_{d}}{L_{d}}\times 1000$$

where *L*(*t*) is the specimen length at immersion time *t*, and *L_d_* is the dry reference length at the corresponding experimental stage, measured along the longitudinal axis of the specimen using a caliper with a resolution of 0.01 mm.

Continuous recording was performed for each of the six specimens after drying at 60 °C and after sequential thermal conditioning at 350 and 500 °C, allowing both the magnitude and temporal evolution of hydric deformation to be compared through successive thermal treatments.

### 2.4. Water Absorption

Water absorption at atmospheric pressure was determined on the same six prismatic specimens used for hydric-deformation measurements. The specimens were fully immersed in distilled water, so water was accessible from all exposed surfaces. The procedure therefore represents a total-immersion water-absorption test rather than a one-dimensional capillary-absorption test through a defined surface. The procedure was adapted to the experimental design from the general principles of EN 13755 [22], because water uptake was determined after the fixed immersion period used for hydric-deformation monitoring rather than after attainment of saturated constant mass. At each experimental stage, the dry specimen mass measured after cooling was used as the reference mass (*m_d_*). The wet mass (*m_w_*) was determined after approximately 8400 min of immersion. Each specimen was removed from the water, rapidly wiped with a damp cloth to remove visible surface water, and weighed within 1 min. Mass measurements were performed using a Mettler Toledo PB4002-S/FACT balance with a readability of 0.01 g (Mettler-Toledo GmbH, Greifensee, Switzerland). Water absorption after 8400 min (*A_W_*) was calculated as

$$A_{W}=\frac{m_{w}-m_{d}}{m_{d}}\times 100$$

and expressed as wt.%. Because *A_W_* was calculated as the percentage increase in specimen mass relative to the corresponding dry mass, specimen cross-sectional area does not enter directly into the calculation. Area normalization would instead be required for capillary-absorption tests in which water enters through a defined exposed surface. Using the same specimens and the same immersion period for water absorption and hydric-deformation measurements allowed water uptake to be directly compared with the corresponding dimensional response.

### 2.5. Ultrasonic Pulse Velocity

Ultrasonic pulse velocity was measured on the same prismatic specimens using a Pundit 200 ultrasonic testing system (Proceq SA, Schwerzenbach, Switzerland) equipped with 54 kHz exponential transducers. Measurements were performed following an internal procedure adapted from EN 14579 [23]. A direct-transmission configuration was used, with the transmitting and receiving transducers positioned on the opposite end faces of each specimen so that the pulse propagated along its longitudinal axis. Dry acoustic contact was established by pressing the transducers firmly against the flat sawn surfaces; no coupling medium was required. The specimen length was measured before each test using a caliper with a resolution of 0.01 mm. The transit-time resolution of the instrument was 0.1 μs. P-wave velocity was calculated from the measured specimen length, L, and pulse transit time, t, as V_P_ = L/t. Ultrasonic measurements were performed after cooling in the silica-gel desiccator and before the subsequent water-absorption and hydric-deformation measurements.

### 2.6. Anisotropy

Directional anisotropy was quantified using the ratio between the larger and smaller mean directional values so that A ≥ 1. For hydric deformation, because HD_MTO_ > HD_MTP_ at all conditioning stages, the anisotropy ratio was calculated as: A_HD_ = HD_MTO_/HD_MTP_.

For ultrasonic pulse velocity, because V_P MTP_ > V_P MTO_, the anisotropy ratio was calculated as: AV_P_ = V_P MTP_/V_P MTO_.

The resulting hydric-deformation anisotropy ratios were 1.24, 1.38, and 1.72 at 60, 350, and 500 °C, respectively, whereas the corresponding ultrasonic-velocity anisotropy ratios were 1.10, 1.11, and 1.13.

### 2.7. Data Treatment

For each bedding orientation and conditioning state, individual values and mean ± standard deviation are reported for three specimens (n = 3). Given the limited number of independent specimens, the analysis was treated as descriptive. Temperature-related changes were evaluated as within-specimen trajectories, with absolute and percentage changes at 350 and 500 °C calculated relative to the corresponding 60 °C reference value for each specimen. Time-series acquisition points were not treated as independent replicates. Given the small number of independent specimens per orientation, no formal null-hypothesis significance testing or standardized effect-size estimation was performed. The analysis therefore emphasizes individual specimen responses, paired changes, group mean ± standard deviation, and the observed experimental variability.

## 3. Results

The specimen-level endpoint dataset is summarized in Table 1.

### 3.1. Continuous Hydric Deformation

Continuous dilatometric measurements showed a systematic decrease in hydric deformation with increasing thermal-conditioning temperature for both bedding orientations.

**Figure 2:**
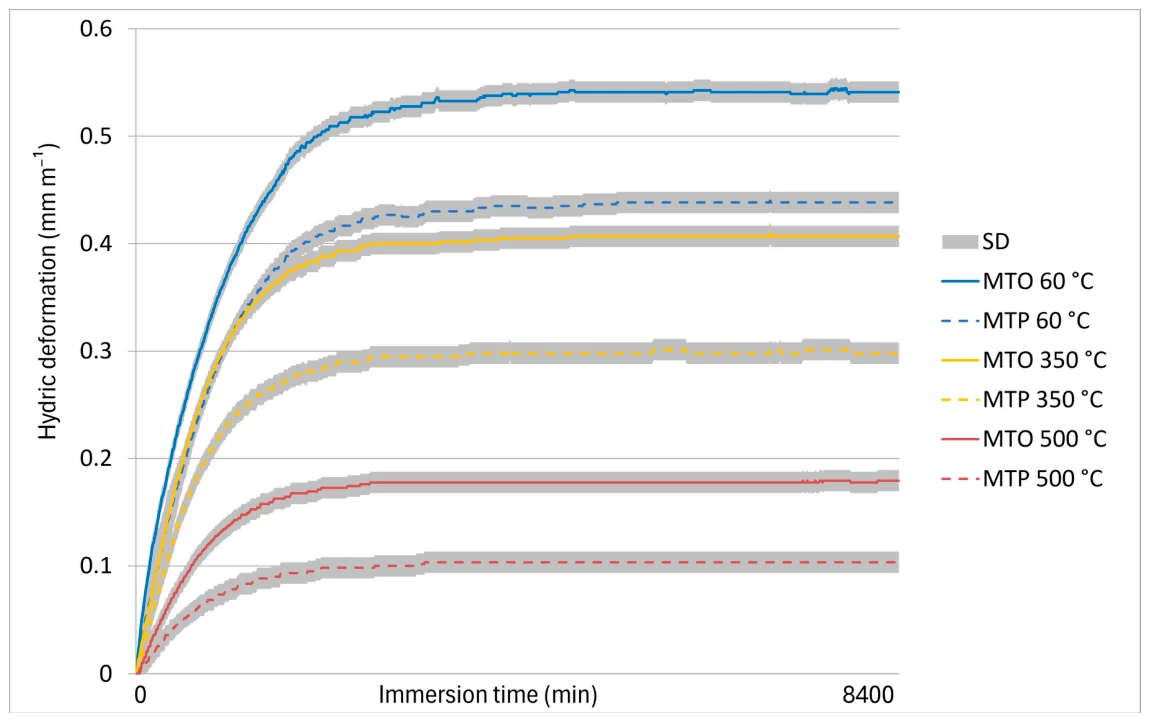
Evolution of hydric deformation with immersion time for Macigno sandstone specimens oriented orthogonal to bedding (MTO) and parallel to bedding (MTP), measured after drying at 60 °C and after sequential thermal conditioning at 350 and 500 °C. Each curve represents the mean hydric deformation of three specimens (n = 3), with colored bands representing the standard deviation calculated at each measurement point.

Figure 2 shows the continuous hydric-deformation curves for MTO and MTP specimens after drying at 60 °C and after sequential thermal conditioning at 350 and 500 °C.

In the reference condition at 60 °C, specimens oriented orthogonal to bedding (MTO) exhibited greater hydric deformation than specimens oriented parallel to bedding (MTP). Mean final deformation values were 0.54 ± 0.01 mm m^−1^ for MTO and 0.44 ± 0.01 mm m^−1^ for MTP.

After sequential thermal conditioning at 350 °C, mean hydric deformation decreased to 0.41 ± 0.01 mm m^−1^ for MTO and 0.30 ± 0.01 mm m^−1^ for MTP. A further marked reduction was observed after treatment at 500 °C, when mean values reached 0.18 ± 0.01 mm m^−1^ and 0.10 ± 0.01 mm m^−1^, respectively.

Relative to the reference condition, hydric deformation decreased by approximately 25% for MTO and 32% for MTP after conditioning at 350 °C. After conditioning at 500 °C, the corresponding reductions were approximately 67% and 76%.

The directional relationship was preserved throughout the complete conditioning sequence, with deformation consistently higher orthogonal to bedding than parallel to bedding. Despite the progressive decrease in absolute deformation, replicate specimens showed limited dispersion at all three conditioning stages.

The continuously recorded deformation curves further allowed the evolution of strain with immersion time to be followed for each specimen and conditioning state. In all cases, hydric deformation increased rapidly during the initial stage of immersion and subsequently approached a near-stable value more gradually. Sequential thermal conditioning markedly reduced the magnitude of the deformation response, while the overall temporal pattern of rapid initial expansion followed by progressive stabilization was broadly maintained at all conditioning temperatures.

The mean kinetic parameters derived from the individual deformation curves are summarized in Table 2. Thermal conditioning produced a systematic reduction in the initial deformation rate in both bedding orientations, whereas the characteristic times t50 and t90 showed comparatively smaller variations.

### 3.2. Water Absorption

Water absorption evolved in the opposite direction to hydric deformation.

In the reference condition, mean water absorption was nearly identical for the two orientations, with values of 0.42 ± 0.01 wt.% for MTO and 0.42 ± 0.01 wt.% for MTP.

After conditioning at 350 °C, water absorption increased to 0.50 ± 0.01 wt.% for MTO and 0.51 ± 0.01 wt.% for MTP. Following treatment at 500 °C, mean values further increased to 0.59 ± 0.01 wt.% and 0.58 ± 0.01 wt.%, respectively.

Thus, relative to the reference condition, water absorption increased by approximately 20% after conditioning at 350 °C and by about 38–41% after conditioning at 500 °C.

Unlike hydric deformation, water absorption showed no consistent bedding-related directional dependence. At all three conditioning stages, the differences between MTO and MTP were small relative to the strong differences observed in dimensional response.

### 3.3. Ultrasonic Pulse Velocity

Ultrasonic pulse velocity decreased progressively with increasing thermal-conditioning temperature, although the magnitude of this change was considerably smaller than that observed for hydric deformation.

For MTO specimens, mean V_P_ decreased from 4347 ± 40 m s^−1^ at 60 °C to 4143 ± 21 m s^−1^ at 350 °C and 3915 ± 19 m s^−1^ at 500 °C. These values correspond to decreases of approximately 5% and 10%, respectively, relative to the reference condition.

For MTP specimens, mean V_P_ decreased from 4770 ± 36 m s^−1^ to 4610 ± 65 m s^−1^ and 4437 ± 103 m s^−1^ over the same conditioning sequence, corresponding to reductions of approximately 3% at 350 °C and 7% at 500 °C.

At each conditioning stage, V_P_ was higher for specimens measured parallel to bedding than for those measured orthogonal to bedding. The dispersion of the MTP measurements increased with treatment temperature, whereas MTO values remained comparatively tightly grouped.

### 3.4. Coupled Evolution of Hydric Deformation, Water Absorption, and Ultrasonic Pulse Velocity

The three measured parameters showed clearly different responses along the sequential thermal-conditioning pathway.

**Figure 3:**
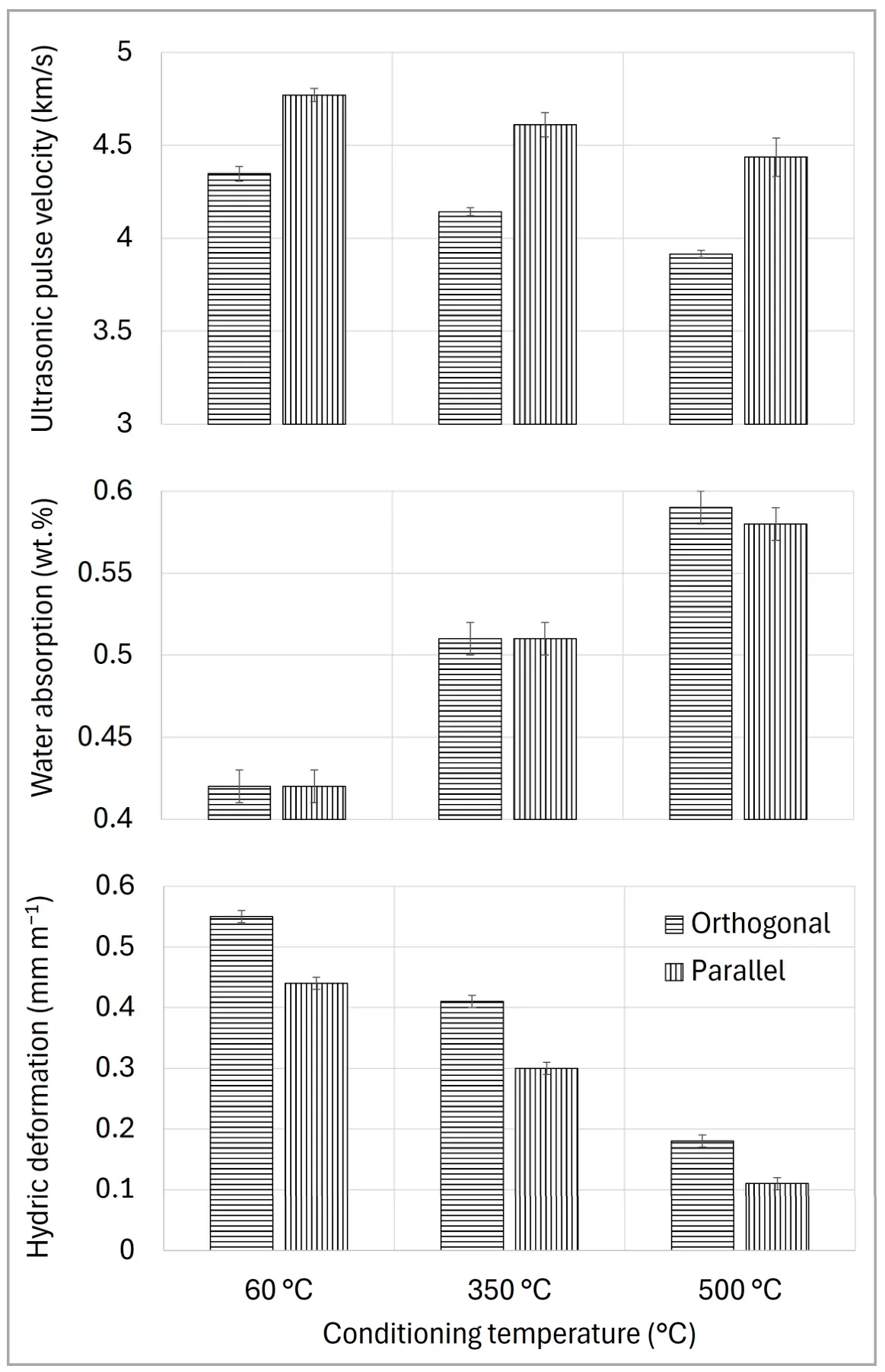
Ultrasonic pulse velocity (V_P_), water absorption (A_W_), and hydric deformation (HD) of Macigno sandstone specimens after drying at 60 °C and after sequential thermal conditioning at 350 and 500 °C. Results are shown separately for specimens oriented orthogonal (MTO) and parallel (MTP) to bedding. Error bars represent the standard deviation (n = 3).

Figure 3 highlights the contrasting responses to sequential thermal conditioning, with a moderate decrease in ultrasonic pulse velocity, an increase in water absorption, and a pronounced reduction in hydric deformation.

From 60 to 500 °C, water absorption increased in both orientations, whereas hydric deformation decreased strongly. Ultrasonic pulse velocity also decreased, but substantially less than hydric deformation.

For MTO specimens, mean water absorption increased from 0.42 to 0.59 wt.% while hydric deformation decreased from 0.54 to 0.18 mm m^−1^. Over the same interval, V_P_ decreased from 4347 to 3915 m s^−1^.

A similar pattern was observed for MTP specimens: water absorption increased from 0.42 to 0.58 wt.%, hydric deformation decreased from 0.44 to 0.10 mm m^−1^, and V_P_ decreased from 4770 to 4437 m s^−1^.

The most prominent feature of the dataset is therefore the opposite evolution of water uptake and hydric deformation. Increasing thermal-conditioning temperature resulted in progressively greater water absorption but markedly lower dimensional response. This behavior was observed consistently in all six specimens and for both bedding orientations.

**Figure 4:**
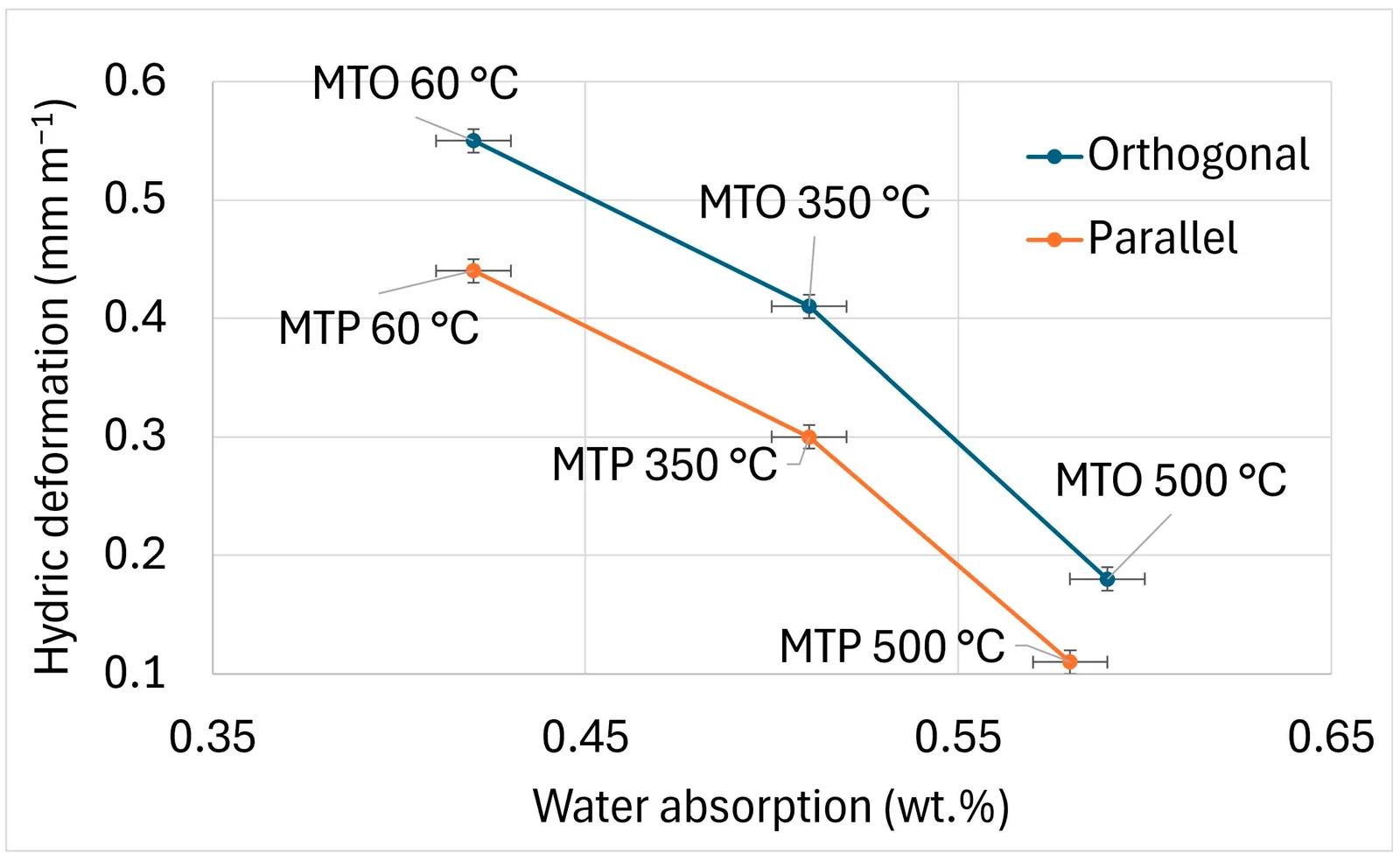
Relationship between water absorption (A_W_) and final hydric deformation (HD) for Macigno sandstone specimens after drying at 60 °C and after sequential thermal conditioning at 350 and 500 °C. MTO and MTP indicate specimens oriented orthogonal and parallel to bedding, respectively. Increasing thermal-conditioning temperature is associated with higher water absorption and lower hydric deformation, indicating an inverse evolution of the two parameters. Error bars represent the standard deviation of water absorption and hydric deformation (n = 3).

The inverse relationship between water absorption and hydric deformation is further illustrated in Figure 4. With increasing thermal-conditioning temperature, both MTO and MTP specimens progressively shift toward higher water absorption and lower hydric deformation.

## 4. Discussion

### 4.1. Bedding Control on Hydric Deformation

Hydric deformation was consistently greater normal to bedding than parallel to bedding throughout the complete conditioning sequence. In the reference state, mean final deformation was 0.54 mm m^−1^ for MTO specimens and 0.44 mm m^−1^ for MTP specimens, and the same directional relationship persisted after conditioning at 350 and 500 °C.

This behavior is consistent with the mineralogical and textural characteristics of Macigno sandstone. Previous studies have documented illite, chlorite, chlorite-smectite mixed-layer phases, and locally corrensite within the phyllosilicate fraction [8,9]. In the investigated material, phyllosilicate-rich domains are preferentially oriented parallel to bedding. Hydration-related expansion within such domains may therefore generate a larger bulk strain normal to the sedimentary fabric.

The observed behavior is also consistent with studies showing that clay-related swelling in sandstone depends not only on total clay content but also on clay mineralogy and its distribution within the rock framework [5,10]. Wangler and Scherer [5] demonstrated that intracrystalline swelling can be the dominant swelling mechanism in clay-bearing sandstones, while Jiménez-González et al. [10] emphasized the importance of clay-rich cementing domains in controlling deformation and deterioration during moisture cycling.

The magnitude of hydric deformation measured in the present Macigno sandstone, approximately 0.44–0.54 mm m^−1^ in the reference condition, is therefore consistent with the moderate moisture-induced dimensional response expected for a compact sandstone containing a limited but mineralogically significant phyllosilicate fraction. More importantly, the systematically larger deformation normal to bedding agrees with previous observations that moisture expansion in sandstones and other layered stones may exhibit pronounced directional dependence associated with the preferred orientation and spatial distribution of clay-bearing domains [7,9].

In contrast to hydric deformation, water absorption showed little systematic dependence on bedding orientation. The near-identical A_W_ values for MTO and MTP therefore indicate that directional deformation cannot be explained simply by differences in the total amount of water entering the specimens. Bedding orientation appears to control how absorbed water produces deformation more strongly than how much water is absorbed. The magnitude of the directional contrast can also be expressed by the ratio between mean MTO and MTP hydric deformation. The HD anisotropy ratio (MTO/MTP) increased from approximately 1.24 at 60 °C to 1.38 at 350 °C and 1.72 at 500 °C. Thus, although the absolute deformation decreased markedly, the relative directional contrast became progressively stronger. By comparison, the corresponding VP anisotropy ratio (MTP/MTO) changed only from approximately 1.10 to 1.11 and 1.13 over the same sequence, indicating a much more modest evolution of acoustic anisotropy.

The persistence of this directional contrast after thermal conditioning therefore indicates that the bedding-related control observed in the reference state is not removed by heating; rather, the relative anisotropy of hydric deformation becomes more pronounced as the absolute deformation decreases.

### 4.2. Progressive Reduction in Hydric Deformation After Thermal Conditioning

Along the sequential conditioning pathway, hydric deformation decreased markedly and systematically in both orientations. Relative to the 60 °C reference state, final deformation decreased by approximately 25% for MTO and 32% for MTP after conditioning at 350 °C, and by approximately 67% and 76%, respectively, after conditioning at 500 °C.

The magnitude of this reduction is noteworthy when compared with previous studies of thermally treated sandstones, which commonly report progressive changes in pore structure, grain contacts, and crack development with increasing temperature [12,13]. The present results extend these observations by showing that such thermal conditioning can be accompanied by a very large reduction in the subsequent moisture-induced dimensional response, reaching approximately 67–76% at 500 °C, even though water absorption increases and ultrasonic pulse velocity decreases much more moderately. Micro-computed tomography observations of thermally treated sandstone have directly documented modification of internal pore and fracture structures with increasing temperature [13].

However, the present experiments do not provide direct microscopic observations of the conditioned Macigno specimens. Consequently, specific mechanisms such as thermal microcracking should not be treated as directly demonstrated here. The experimental evidence supports thermal modification of the stone fabric, whereas the precise pore-scale processes responsible for the large reduction in hydric deformation require dedicated post-treatment microstructural characterization.

The two conditioning intervals should nevertheless be considered separately. Between 60 and 350 °C, the observed changes may reflect progressive removal of retained water, differential thermal expansion among mineral constituents, modification of grain contacts, and initiation or opening of pre-existing discontinuities. Between 350 and 500 °C, differential thermal expansion and thermally induced microcracking may become more pronounced, while some phyllosilicate phases may begin to undergo structural modification or partial dehydroxylation depending on their mineralogical composition. Because no post-treatment XRD, TGA, or equivalent mineralogical characterization was performed, these processes should be regarded as possible mechanisms rather than directly demonstrated transformations in the investigated specimens.

Continuous dilatometric acquisition is particularly relevant because it provides the complete evolution of deformation during immersion rather than a single endpoint value. The curves show that the marked reduction in final deformation after sequential thermal conditioning occurs while the general temporal pattern of the hydric response (rapid initial expansion followed by a progressively slower approach to stabilization) is broadly preserved. Continuous acquisition therefore provides information on the temporal development of hydric deformation that is not available from conventional endpoint measurements.

The kinetic analysis further refines this observation. Sequential thermal conditioning markedly reduced the early deformation rate, whereas t50 and t90 remained of the same general order of magnitude among the conditioning states. This indicates that heating primarily affected the magnitude and early intensity of the hydric response, rather than producing a systematic slowing of the normalized approach to the near-stable deformation.

### 4.3. Inverse Evolution of Water Uptake and Hydric Deformation

The most prominent result is the inverse evolution of water absorption and hydric deformation with increasing conditioning temperature.

Between 60 and 500 °C, mean water absorption increased from 0.42 to 0.59 wt.% for MTO specimens and from 0.42 to 0.58 wt.% for MTP specimens. Over the same interval, hydric deformation decreased from 0.54 to 0.18 mm m^−1^ and from 0.44 to 0.10 mm m^−1^, respectively.

Thus, greater water uptake after heating is associated with lower, rather than higher, dimensional deformation. This inverse evolution is directly supported by the experimental measurements. Its underlying microstructural causes, however, cannot be established from the present dataset because no post-treatment mineralogical or microstructural characterization was performed.

This observation is important because hydric deformation in clay-bearing sandstone cannot be considered simply proportional to the total mass of absorbed water. Clay-swelling studies show that deformation depends on water-clay interactions, the nature of swelling-active phases, and their location within the rock framework [5,10]. The additional water absorbed after sequential thermal conditioning may therefore occupy newly accessible pore or discontinuity space without contributing proportionally to hydration-related expansion.

One possible interpretation is that heating progressively increases water-accessible volume while reducing the proportion of absorbed water that interacts effectively with deformation-active phyllosilicate domains. A second possibility is that sequential thermal conditioning changes the subsequent hydration response of the clay-bearing domains themselves. The presence of chlorite-smectite mixed-layer phases in Macigno sandstone makes such an effect plausible [8,9], but it cannot be demonstrated from the present dataset because no post-treatment clay-mineral characterization was performed.

Water–mineral interaction during immersion may modify the hydration state of clay-bearing domains and thereby affect dimensional strain without necessarily producing a change in the bulk mineralogical assemblage. Conversely, dissolution, irreversible mineral transformation, or the formation of new mineral phases cannot be assessed from the present dataset because no mineralogical characterization was performed before and after the immersion sequence.

The results therefore support a distinction between water-accessible pore space and hydric deformation capacity. This distinction is consistent with the broader understanding that thermo-hydric alteration can modify transport and dimensional properties through different and partially independent mechanisms [11].

### 4.4. Ultrasonic Response as a Complementary Indicator

Ultrasonic pulse velocity also decreased systematically with increasing conditioning temperature, although the magnitude of the change was considerably smaller than that observed for hydric deformation.

Mean V_P_ decreased by approximately 5% at 350 °C and 10% at 500 °C for MTO specimens and by approximately 3% and 7%, respectively, for MTP specimens. The progressive decrease indicates a modification of the elastic transmission properties of the sandstone and is consistent with thermally induced changes in grain contacts and internal discontinuities reported for heated sandstones [12,13].

The V_P_ data should nevertheless be interpreted cautiously. Ultrasonic measurements provide an indirect indication of changes in elastic continuity but do not identify the geometry or origin of the responsible microstructural features. Direct investigations such as micro-computed tomography have demonstrated the development and evolution of thermally induced discontinuities in other sandstones [13], but comparable observations were not performed on the Macigno specimens investigated here.

An important feature of the present dataset is that the decrease in V_P_ is relatively moderate compared with the very large reduction in hydric deformation. Hydric deformation therefore does not simply scale with ultrasonic pulse velocity. Instead, the three parameters describe complementary aspects of thermal modification: water absorption reflects water accessibility, V_P_ reflects changes in elastic transmission, and HD reflects the ability of the stone to convert hydration processes into macroscopic dimensional strain.

The large discrepancy between the evolution of HD and VP indicates that these parameters have different sensitivities to thermal modification. Ultrasonic pulse velocity integrates elastic transmission through the bulk grain-contact network and is affected by discontinuities along the propagation path. Hydric deformation, in contrast, is more directly related to the capacity of moisture-responsive mineral domains to convert water–mineral interactions into macroscopic strain. A thermal modification affecting the effectiveness or connectivity of a relatively small fraction of deformation-active phyllosilicate-rich domains could therefore strongly reduce HD without producing a proportional decrease in bulk VP.

### 4.5. Value of Continuous Monitoring and Repeated Measurements

An important limitation of the experimental design is that the same specimens were subjected sequentially to water immersion, re-drying, and heating. The three conditioning states are therefore not independent, and the observed changes describe the cumulative 60–350–500 °C experimental pathway. Because no separate control set underwent repeated immersion and drying without heating, a contribution from prior wetting-drying history or sequence effects cannot be isolated from the effect associated with increasing treatment temperature. The results should consequently be interpreted as within-specimen trajectories under the sequential protocol used here.

This approach is particularly useful for natural stone, where intrinsic mineralogical and textural heterogeneity may otherwise obscure relatively small treatment-related changes. Hydric-deformation measurements showed limited dispersion among the three replicate specimens within each orientation.

Continuous monitoring provides an additional advantage. Conventional water-absorption measurements describe the amount of water taken up, while endpoint dilatometric measurements provide only the final dimensional response. Continuous acquisition preserves the temporal information contained in the deformation process, allowing the initial response, subsequent evolution, and approach to stabilization to be compared among conditioning states. The kinetic information obtained from continuous monitoring represents an additional advantage over conventional endpoint measurements. Two specimens may exhibit similar final deformation while reaching that state at substantially different rates. Parameters such as the initial deformation rate and characteristic times therefore provide information on the dynamics of water-rock interaction that cannot be recovered from a single endpoint measurement.

The combination of repeated measurements and continuous acquisition therefore provides a compact framework for distinguishing changes in water uptake from changes in deformation capacity in thermally conditioned sandstone.

### 4.6. Implications for Durability Assessment

The strong reduction in hydric deformation after sequential thermal conditioning should not be interpreted as evidence of improved durability. The simultaneous increase in water absorption and decrease in ultrasonic pulse velocity indicate that the stone undergoes substantial thermal modification despite its lower dimensional response. These results highlight the need to consider water uptake, ultrasonic response, and hydric deformation as complementary indicators when assessing thermally modified sandstone. Likewise, a reduction in hydric deformation does not necessarily indicate preservation of the original microstructure.

## 5. Conclusions

Continuous dilatometric monitoring was used to follow the hydric deformation of six Macigno sandstone prisms, comprising three specimens oriented orthogonal to bedding (MTO) and three parallel to bedding (MTP), in the reference state after drying at 60 °C and after sequential thermal conditioning at 350 and 500 °C.

The repeated-measurement approach showed a clear and progressive modification of the hydric response with increasing conditioning temperature.

In the reference condition, hydric deformation was systematically greater normal to bedding than parallel to bedding, with mean values of 0.54 and 0.44 mm m^−1^, respectively. This directional relationship was maintained after sequential thermal conditioning, indicating a persistent control of the sedimentary fabric on deformation.

Along the sequential conditioning pathway, hydric deformation decreased markedly. After treatment at 500 °C, mean deformation decreased to 0.18 mm m^−1^ for MTO specimens and 0.10 mm m^−1^ for MTP specimens, corresponding to reductions of approximately 67% and 76% relative to the reference condition.

Water absorption showed the opposite trend, increasing from approximately 0.42 wt.% to 0.58–0.59 wt.% between the reference state and 500 °C. The results therefore demonstrate a clear inverse evolution of water absorption and hydric deformation under the adopted sequential conditioning protocol: increased water absorption after thermal conditioning was accompanied by lower dimensional response.

Ultrasonic pulse velocity decreased progressively but more moderately, by approximately 7–10% at 500 °C depending on bedding orientation. This evolution is consistent with progressive thermal modification of the stone fabric, although the specific microstructural mechanisms responsible for the changes require direct post-treatment characterization.

The combined results show that hydric deformation, water absorption, and ultrasonic pulse velocity describe different aspects of the response of thermally conditioned sandstone. In particular, water absorption alone cannot be used as an indicator of hydric deformation capacity.

Continuous monitoring of the same specimens through successive conditioning stages provides a useful approach for detecting changes in both the magnitude and temporal evolution of moisture-induced deformation while minimizing the influence of specimen-to-specimen variability.

From a practical perspective, the marked reduction in hydric deformation after thermal conditioning should not be interpreted as evidence of material recovery or improved durability, because it occurs together with increased water accessibility and reduced ultrasonic pulse velocity.

## Figures and Tables

**Table 1:** Ultrasonic pulse velocity (V_P_), water absorption (A_W_), and final hydric deformation (HD) measured on the six Macigno sandstone prismatic specimens after drying at 60 °C and after sequential thermal conditioning at 350 and 500 °C. MTO indicates specimens oriented orthogonal to bedding and MTP specimens oriented parallel to bedding. Mean values and standard deviations are reported for each orientation (n = 3).

Sample	V_P_ 60 °C	V_P_ 350 °C	V_P_ 500 °C	A_W_ 60 °C	A_W_ 350 °C	A_W_ 500 °C	HD 60 °C	HD 350 °C	HD 500 °C
	(m s^−1^)	(m s^−1^)	(m s^−1^)	(wt.%)	(wt.%)	(wt.%)	(mm m^−1^)	(mm m^−1^)	(mm m^−1^)
MTO-1	4301	4122	3912	0.42	0.50	0.59	0.55	0.42	0.19
MTO-2	4374	4163	3935	0.42	0.50	0.60	0.54	0.40	0.18
MTO-3	4367	4145	3897	0.42	0.51	0.59	0.54	0.41	0.18
Mean MTO	4347	4143	3915	0.42	0.50	0.59	0.54	0.41	0.18
St. Dev.	40	21	19	0.01	0.01	0.01	0.01	0.01	0.01
MTP-1	4786	4674	4545	0.42	0.52	0.58	0.44	0.30	0.11
MTP-2	4795	4612	4424	0.43	0.50	0.59	0.43	0.29	0.10
MTP-3	4728	4544	4341	0.42	0.51	0.58	0.44	0.30	0.11
Mean MTP	4770	4610	4437	0.42	0.51	0.58	0.44	0.30	0.10
St. Dev.	36	65	103	0.01	0.01	0.01	0.01	0.01	0.01

**Table 2:** Mean kinetic parameters derived from the continuous hydric-deformation curves of Macigno sandstone specimens. Values are reported as mean ± standard deviation for three specimens (n = 3) for each bedding orientation and conditioning state. HDfinal is the mean deformation over the final 30 min of immersion; the initial deformation rate was calculated by linear regression over the first 120 min; t50 and t90 are the times required to reach 50% and 90% of HDfinal, respectively.

Sample	Conditioning Temperature	HDfinal (mm m^−1^)	Initial Deformation Rate (mm m^−1^ min^−1^)	t50 (min)	t90 (min)
MTO	60 °C	0.541 ± 0.007	(7.05 ± 0.82) × 10^−4^	597 ± 17	1802 ± 59
MTP	60 °C	0.438 ± 0.005	(4.70 ± 2.02) × 10^−4^	604 ± 44	1789 ± 116
MTO	350 °C	0.407 ± 0.008	(4.34 ± 0.22) × 10^−4^	527 ± 10	1569 ± 52
MTP	350 °C	0.298 ± 0.005	(3.27 ± 0.13) × 10^−4^	500 ± 9	1563 ± 50
MTO	500 °C	0.179 ± 0.006	(1.85 ± 0.02) × 10^−4^	529 ± 15	1558 ± 120
MTP	500 °C	0.103 ± 0.003	(1.10 ± 0.10) × 10^−4^	530 ± 23	1683 ± 254

## Data Availability

The original contributions presented in this study are included in the article. Further inquiries can be directed to the corresponding author.
